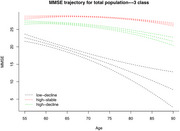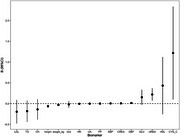# Composite phenotypes and multiple organ systems aging clocks predict cognitive decline

**DOI:** 10.1002/alz70857_101774

**Published:** 2025-12-25

**Authors:** Yingzhe Wang

**Affiliations:** ^1^ Huashan Hospital, Fudan University, Shanghai, Shanghai, China

## Abstract

**Background:**

Aging variance has been evaluated using biological age, yet the multiple organ systems aging clocks of cognitive decline warrants further study.

**Method:**

Using brain imaging and physiological phenotypes from the Taizhou Imaging Study and Shanghai Aging Study (*N* = 1448), we examined composite phenotypic aging and subsequently established aging clocks for brain and four body systems.

**Result:**

The study included 1,448 participants from the community, ranging in age from 55 to 92 years. All subjects underwent cognitive assessments at at least three time points. The subjects were divided into three groups according to the cognitive trajectory: low‐decline, high‐decline, high‐stable. Some measures of kidney function, such as creatinine and homocysteine, were significantly associated with cognitive decline. Composite phenotypes such as renal and metabolic altered with cognitive function and could serve as cognitive aging clock features.

**Conclusion:**

Our research uncovers new insights into heterogeneous phenotypic and organ aging, promoting the development of comprehensive and tailored strategies to manage cognitive aging.